# Genome-Wide Identification of Target Genes for the Key B Cell Transcription Factor *Ets1*

**DOI:** 10.3389/fimmu.2017.00383

**Published:** 2017-04-07

**Authors:** Prontip Saelee, Alyssa Kearly, Stephen L. Nutt, Lee Ann Garrett-Sinha

**Affiliations:** ^1^Department of Biochemistry, State University of New York at Buffalo, Buffalo, NY, USA; ^2^The Walter and Eliza Hall Institute of Medical Research, Parkville, VIC, Australia; ^3^Department of Medical Biology, University of Melbourne, Parkville, VIC, Australia

**Keywords:** Ets1, target genes, ChIP-seq, RNA-seq, promoter, enhancer, autoimmunity

## Abstract

**Background:**

The transcription factor Ets1 is highly expressed in B lymphocytes. Loss of Ets1 leads to premature B cell differentiation into antibody-secreting cells (ASCs), secretion of autoantibodies, and development of autoimmune disease. Despite the importance of Ets1 in B cell biology, few Ets1 target genes are known in these cells.

**Results:**

To obtain a more complete picture of the function of Ets1 in regulating B cell differentiation, we performed Ets1 ChIP-seq in primary mouse B cells to identify >10,000-binding sites, many of which were localized near genes that play important roles in B cell activation and differentiation. Although Ets1 bound to many sites in the genome, it was required for regulation of less than 5% of them as evidenced by gene expression changes in B cells lacking Ets1. The cohort of genes whose expression was altered included numerous genes that have been associated with autoimmune disease susceptibility. We focused our attention on four such Ets1 target genes Ptpn22, Stat4, Egr1, and Prdm1 to assess how they might contribute to Ets1 function in limiting ASC formation. We found that dysregulation of these particular targets cannot explain altered ASC differentiation in the absence of Ets1.

**Conclusion:**

We have identified genome-wide binding targets for Ets1 in B cells and determined that a relatively small number of these putative target genes require Ets1 for their normal expression. Interestingly, a cohort of genes associated with autoimmune disease susceptibility is among those that are regulated by Ets1. Identification of the target genes of Ets1 in B cells will help provide a clearer picture of how Ets1 regulates B cell responses and how its loss promotes autoantibody secretion.

## Introduction

B cells are crucial contributors to immunity both by secreting specific antibodies and by serving as antigen-presenting cells. B cell immune responses and differentiation to antibody-secreting cells (ASCs) are controlled by the expression and activation of specific transcription factors at different stages and time points in B cell development and functional activation. For instance, the transcription factors Pax5, Ebf1, E2a (Tcf3), and Foxo1 are required for the development of B cell progenitors in the bone marrow and in mature and peripheral B cells (1–7). A different set of transcription factors including Blimp1 (Prdm1), Xbp1, and Irf4 are required for B cells to undergo ASC differentiation. Blimp1 stimulates immunoglobulin secretion and suppresses mature B cell gene expression (8, 9). Xbp1 is required for endoplasmic reticulum remodeling and high-level immunoglobulin production (10, 11). Irf4 is also required for ASC differentiation (12), although the mechanisms by which it does so are still under debate (13).

In addition to the transcription factors summarized above, various members of the Ets gene family also regulate B cell differentiation and function. The roles of six Ets proteins have been studied in detail in B lymphocytes, including Ets1 (14), Fli1 (15, 16), Gabpa (17), and the three related Ets family factors PU.1, SpiB, and SpiC (18, 19). Two additional Ets proteins, Elf3 (Ese1) and Elf4 (MEF) have been reported to affect B cell development or functional responses (20, 21), although their detailed roles in this process have not yet been identified.

Ets1, the prototypical member of the Ets gene family, is highly expressed in B cells and is necessary for maintaining them in a quiescent state, since lack of Ets1 leads to premature differentiation into ASCs (22, 23). This is accompanied by a loss of B cell tolerance to self-antigens (24). *Ets1^−/−^* mice also lack marginal zone type B cells, possibly because of depletion due to excessive differentiation to ASCs (23, 25). In keeping with a role for Ets1 in establishing B cell tolerance, *Ets1^−/−^* mice develop an autoimmune phenotype (23, 26) and single-nucleotide polymorphisms (SNPs) in the human *ETS1* gene have been highly implicated in a variety of autoimmune diseases (27, 28).

The most well-studied function of Ets1 in B cells is in regulating the formation of ASCs. One mechanism by which Ets1 regulates this process is by forming a protein–protein complex with Blimp1 resulting in the inhibition of Blimp1 DNA binding (22, 29). Ets1 may also regulate B cell differentiation through direct binding to target genes. To date, only a few such target genes of Ets1 have been identified in B cells, including *Pax5* (22, 29–37), which is crucial for maintaining mature B cell identity. In our study, we identify Ets1-binding sites in mouse B cells using ChIP-sequencing and identify gene expression changes in the absence of Ets1 using RNA-sequencing. Interestingly, many of these target genes are implicated in autoimmune responses, a cohort of which is tested for their ability to restore normal differentiation to *Ets1^−/−^* B cells. Restoring the normal expression levels of four of these Ets1 targets (Stat4, Ptpn22, Egr1 and Prdm1) failed to reverse the *Ets1^−/−^* B cell phenotype of excessive plasma cell differentiation in response to TLR ligands. Instead, restoring Stat4 and Ptpn22 resulted in increased plasma cell differentiation. Therefore, other targets of Ets1 or the combined actions of multiple targets may be crucial for regulating this B cell differentiation step.

## Materials and Methods

### Mice

Wild-type (WT) C57BL/6 mice were purchased from Jackson Laboratory. *Ets1^−/−^* mice (RRID:MGI: 3833458) and littermate WT controls used in this study were bred in our facility and maintained on a mixed genetic background of C57BL/6 × 129Sv because, on a pure C57BL/6 genetic background, the loss of Ets1 is lethal perinatally. The mutation in the Ets1 locus of these mice has previously been described (23, 38). Mice carrying the Prdm1-green fluorescent protein (GFP) allele that inactivates Blimp1 were obtained from Dr. Stephen Nutt (Walter and Eliza Hall Institute of Medical Research, Parkville, VIC, Australia) (39).

### B Cell Purification and ChIP-seq

Wild-type mouse B cells were purified from spleens of 3-month-old C57BL/6 male mice using negative selection with the Easysep mouse B cell isolation kit (Stem Cell Technologies). Purified B cells were rested for 1 h in complete media [RPMI 1640 + 10% fetal bovine serum, 1% pen/strep, 1% Glutamax (Gibco), and 50 μM β-mercaptoethanol] in a tissue culture incubator. After resting, cells were cross-linked by adding formaldehyde to a final concentration of 0.25% for 8 min. Fixed B cells were lysed and chromatin prepared according to the manufacturer’s protocol for the ChIP-IT High Sensitivity Kit (Active Motif). Chromatin was sonicated to yield fragments of an average size ~200–700 bp and immunoprecipitated with a rabbit polyclonal anti-Ets1 antibody (sc-350X, Santa Cruz) that has previously been used in chromatin immunoprecipitation assays (40–42). Two biological replicates were separately prepared and analyzed. The number of uniquely mapped reads for ChIP-seq was between 10 and 27 million for the Ets1 ChIP-seq and between 7 and 10 million for sequencing of the input.

Libraries were generated from the purified chromatin, and ChIP-sequencing was performed on input chromatin and Ets1-precipitated chromatin using an Illumina Hiseq2500 Sequencing System. The ChIP-seq data were found to be of good quality using the normalized strand coefficient and the relative strand correlation parameters as described previously (43). The reads were aligned to mouse mm9 genome assembly using Bowtie (44).

### Bioinformatics Analyses of ChIP-seq

We identified Ets1-bound regions using the MACS2 program (45). Peaks identified from each biological replicate were compared to input controls using the irreproducible discovery rate to identify reproducible Ets1-binding sites of which 10,391 were detected. Ets1-bound regions were annotated for enrichment at intergenic regions, promoters, exons, or introns using ChIPseeker (46). Overrepresented motifs in the peaks were analyzed using the findmotifgenome function in Homer (47). Locations of binding sites with respect to potential target genes were visualized in GenomeBrowser (https://genome.ucsc.edu/cgi-bin/hgGateway).

Ets1 consensus binding sites and other transcription factor-binding sites in the Ets1-bound regions were identified using PscanChIP (48). The regions were then aligned centered on the Ets1 consensus motif. ChIP-seq data for acetylated and methylated lysine variants of histone H3 in primary B cells were obtained from the GEO database and are reported in Ref. (49–51). The program ArchTex (52) was then used to map average histone modifications surrounding the Ets1 consensus-binding sites. As a control, ArchTex was also used to align histone modifications surrounding 60,000 random consensus Ets1-binding sites from regions not bound by Ets1 in ChIP-seq assays.

To test if Ets1 might co-regulate some of the same genes as Pax5, Tcf3 (E2A), or Irf4, we obtained ChIP-seq data for these factors in primary mouse splenic B cells, as reported in Ref. (51, 53, 54). We used Bedtools to identify the co-occurrence between peaks and common target sites for these transcription factors.

### RNA-seq Analysis

Spleens from female WT (C57BL/6) and Ets1 knockout mice (C57 × 129Sv) were used to prepare single cell suspensions, and quiescent follicular B cells were sorted from each population based on the following markers: B220^+^ CD23^hi^ CD21^low^ CD80^neg^ IgA^neg^ IgE^neg^ IgG1^neg^ IgG2a^neg^ IgG2b^neg^ IgG3^neg^ using FACSAria II cell sorter. Dead cells were gated out from the sorted population using Live/Dead Fixable Aqua dead cell stain (Molecular Probes). Total RNA was isolated from two biological replicates of sorted B cells and subjected to RNA-sequencing on an Illumina Hiseq2500 Sequencing System. Sequence reads (24–49 million per sample) were aligned to the mm9 genome assembly using the Tophat program, and differences in gene expression between samples were analyzed using Cuffdiff (55). For further analysis, we chose genes that showed a fold change between WT and *Ets1^−/−^* B cells of at least twofold [log_2_(FC) = 1.0 or more] and a *q* value of 0.05 or less. We excluded genes with a reported Fragments Per Kilobase of transcript per Million mapped reads (FPKM) of less than 1.0 in either genotype. CummeRbund was used to visualize the Cuffdiff analysis, including generating heat maps of gene expression levels.

Genes that showed an alteration in expression as described above were analyzed for their functions by DAVID software (56) to identify the most-relevant gene ontology (GO) terms that were significantly enriched. We used an EASE score equal to 0.05 and a count threshold of 5 to identify enriched pathways.

### RNA Isolation and Reverse Transcription-PCR

To validate RNA-seq data, we purified B cells from the spleens of 3-month-old male and female WT and *Ets1^−/−^* mice (C57BL/6 × 129Sv genetic background) by first using magnet beads (Stem Cell Technologies mouse B cell purification kit) followed by sorting mature naïve follicular B cell subset B220^+^ CD23^hi^ CD21^low^ CD11b^neg^ CD80^neg^ IgA^neg^ IgE^neg^ IgG1^neg^ IgG2a^neg^ IgG2b^neg^ IgG3^neg^. RNA was extracted from sorted B cells (*n* = 4 of each genotype) using Direct-zol RNA MiniPrep kits (Zymo Research). cDNA was synthesized from equal amounts of RNA using QuantiTect Reverse Transcription kit (Qiagen). Quantitative RT-PCR was performed using iQ SYBR Green Supermix (Bio-Rad). The sequences of primers used for RT-PCR were: *Slamf6* (Forward-CAGCTAATGAATGGCGTTCTAGG, Reverse-CTTAGGTTGATAACGAGGGCAG), *Egr1* (Forward-AACCGGCCCAGCAAGACACC, Reverse-TGGCAAACTTCCTCCCACAAAT), *Ptpn22* (Forward-AGCAAGCCTACAGAACGTG, Reverse-TCCAGAGGTGCGTTACATATTC), *Stat4* (Forward-TGGCAACAATTCTGCTTCAAAAC, Reverse-GAGGTCCCTGGATAGGCATGT), *Prdm1* (Forward-TGTGGTAATGTCGGGACTTTG, Reverse-TTCCTTTTGGAGGGATTGGAG), *Hprt* (Forward-CCTCATGGACTGATTATGGACAG, Reverse-TCAGCAAAGAACTTATAGCCCC), *Gapdh* (Forward-AATGGTGAAGGTCGGTGTG, Reverse-GTGGAGTCATACTGGAACATGTAG), and *Actb* (β-actin) (Forward-GCAGCTCCTTCGTTGCCGGTC, Reverse-TTTGCACATGCCGGAGCCGTTG). Gene expression was normalized to all three housekeeping genes (*Gapdh, Hprt*, and *Actb*) using Bio-Rad CFX Manager Software.

### Retroviral Transduction

Plasmids encoding mouse Stat4 (57) or human myc-tagged Ptpn22 (58) were obtained from Dr. John O’Shea (National Institutes of Health, Bethesda, MD, USA) or Dr. Dimitar Efremov (International Centre for Genetic Engineering and Biotechnology, Rome, Italy), respectively. The cDNAs were cut from the original plasmids and sub-cloned into the MIGR1 retroviral plasmid, which contains an internal ribosomal entry site (IRES) followed by GFP to allow easy identification of virally infected cells. The resulting plasmids were confirmed by sequencing. The MIGR1 plasmid encoding mouse Ets1 has been described previously (22). The plasmid used for Egr1 knockdown was generated by cloning a shRNA against Egr1 into the microRNA-30-adapted shRNAmir retroviral vector (MSCV-lmp) from Open Biosystems. Oligonucleotides with sense and antisense strands of an shRNA targeting nucleotides 2,314–2,336 of mouse Egr1 mRNA (NM_007913.5) were cloned into MSCV-lmp and confirmed by sequencing. MSCV-lmp also contains an IRES-GFP module that can be used for detecting transduction. A retroviral plasmid containing a shRNA against Prdm1 in MSCV-lmp, where the GFP gene was substituted with a GFP variant Ametrine (59), was obtained from Dr. Matthew E. Pipkin (The Scripps Research Institute, Jupiter, FL, USA).

Retroviral plasmids were used to transfect the Plat-E packaging cell line along with the plasmid pCL-Eco (which contains additional copies of the viral structural genes) using Fugene-6. The retroviral supernatant was harvested after 48 h of transfection and used to infect WT or *Ets1^−/−^* B cells that were purified using anti-B220 microbeads (Miltenyi Biotec) and stimulated with 10 μg/ml of lipopolysaccharide (LPS) for 24 h. The infected B cells were subsequently returned to fresh medium containing LPS. Forty-eight hours later, B cells were stained for flow cytometry with antibodies to B220 and CD138. Just prior to flow cytometry, 7AAD was added to allow exclusion of dead cells from analysis. Flow cytometry data were collected on a BD LSR II flow cytometer and analyzed using FlowJo software.

To test viral gene expression, infected cells were harvested to make lysates. For the Stat4-virus infected cells, we detected expression in the packaging cell line Plat-E as well as in sorted GFP^+^ virally-infected B cells. For the other constructs, we used unsorted, infected B cells to make lysates. Protein expression was analyzed by Western blotting using the following monoclonal antibodies: anti-Ptpn22 (clone D6D1H; Cell Signaling Technology), anti-Egr1 (clone T.126.1; Thermo Fisher Scientific), anti-Stat4 (clone C46B10; Cell Signaling Technology), anti-Blimp1 (clone 6D3; EMD Millipore), and anti-GAPDH (clone 6C5; EMD Millipore).

### Statistical Analysis

Statistical analysis for qPCR and ELISPOT assays were performed using GraphPad Prism software. *p*-Value was calculated using unpaired Student’s *t*-test with two-tailed *p-*value or with the Mann–Whitney *U*-test.

## Results

### Identification of Ets1-Binding Sites in the Chromatin of B Cells

**Figure 1:**
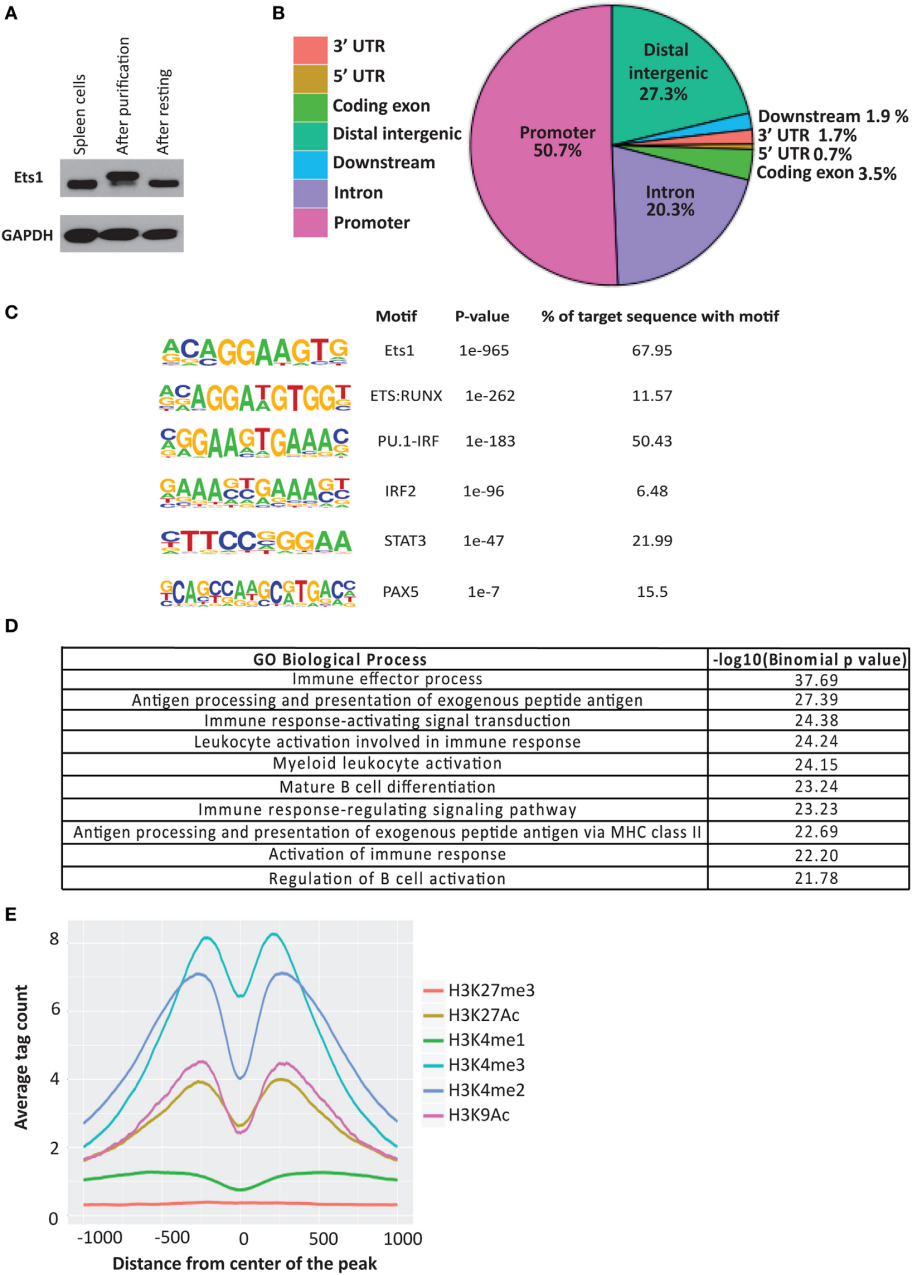
**Identification of Ets1-binding sites in mouse B cells**. **(A)** Western blot to show phosphorylation of Ets1 in freshly isolated B cells versus rested B cells. GAPDH serves as loading control. **(B)** Pie chart of location of Ets1 sites in the genome. **(C)** Motifs enriched in Ets1-bound regions. Shown are overrepresented transcription factor-binding motifs localized in the Ets1 peaks and the percent of sites with that motif. **(D)** Gene ontology biological terms associated with Ets1-binding peaks in B cells. **(E)** Analysis of epigenetic features surrounding Ets1-bound regions by mapping adjacent histone modifications. Data come from the ENCODE Consortium or from the studies described in Ref. (50, 51).

To gain insight into how Ets1 mechanistically regulates B cell differentiation, we assessed its genome-wide occupancy by chromatin immunoprecipitation followed by deep sequencing (ChIP-seq) using chromatin derived from mouse mature B cells. Purified splenic B cells showed enhanced phosphorylation of Ets1 based on SDS-PAGE mobility when compared to whole spleen (Figure 1A). This shift in mobility arises from calcium-induced CAM kinase-dependent serine phosphorylation (34, 60) and results in inhibition of Ets1 DNA binding (61). In order to restore Ets1-binding activity, we rested the B cells for 1–2 h, which resulted in normalization of Ets1 phosphorylation status (Figure 1A). After the resting period, purified B cells were fixed and sonicated.

B cell chromatin was immunoprecipitated with an anti-Ets1 antibody that has been used previously in chromatin immunoprecipitation (40–42). We identified 10,391 reproducible peaks of Ets1 binding, which target 8,975 genes in B cells (GEO dataset: GSE83758). Among Ets1-bound regions, approximately half are in the promoters of the genes (within 3 kb of the transcriptional start site). An additional 20% are in the introns of genes and about 27% are located in distal intergenic regions that may represent enhancers or silencers (Figure 1B). Less than 10% of Ets1-binding sites were detected in coding exons, the 5’ or 3’ UTRs of genes or within 3 kb downstream of target genes. We identified the consensus Ets1-binding motif in 68% of target sites (Figure 1C). Additional transcription factor motifs that were enriched in the Ets1-bound regions include combined ETS-RUNX sites, combined PU.1-IRF sites, IRF2 sites, STAT3 sites, and PAX5 sites (Figure 1C).

Pax5 is a key transcription factor that regulates B cell identity and limits differentiation to ASCs (62, 63). We have previously implicated Ets1 in controlling Pax5 levels in B cells (22, 29). Indeed, examination of the ChIP-seq peaks of Ets1 showed several peaks in the *Pax5* gene including one in the proximal promoter and several in introns of the gene including two strong peaks in intron 5 where a B cell-specific enhancer of *Pax5* has been described (64) (Figure S1 in Supplementary Material). These observations support the idea that Ets1 may directly regulate expression of the *Pax5* gene. Several strong Ets1-binding peaks were also detected near the *Prdm1* gene, which encodes Blimp1 (Figure S2 in Supplementary Material). Although these regions are not associated with any known regulatory elements, they are enriched in H3K4me1 and H3K27Ac marks indicative that they may represent functional regulatory elements. When examining other genes that have been previously described as Ets1 targets [such as those encoding *Cd79a* (Igα), *H-2Aa* and *H-2Eb1* (isoforms of MHC II), and *Nfkb1* (p50)], we found that each gene contained nearby Ets1-binding sites (Figure S3 in Supplementary Material) and, therefore, is potentially regulated by Ets1 *in vivo*.

### Analysis of Patterns of Ets1 Binding in B Cells

To understand how Ets1 might regulate B cell responses, we analyzed pathways associated with Ets1 binding. Ets1-binding peaks were enriched in genes associated with immune response, antigen processing and presentation, immune signaling pathways, and mature B cell differentiation (Figure 1D). Interestingly, Ets1-binding peaks are located near a large number of genes involved in the BCR signaling cascade (Figure S4 in Supplementary Material). Additional non-immune pathways enriched in Ets1-binding sites included genes involved in processing of non-coding RNA, protein folding, and apoptosis (data not shown).

The ENCODE project has mapped histone modifications and transcription factor-binding sites for various human and mouse cell lines (65, 66). In mature primary mouse B cells, data are available for the H3K27me3 modification (49). In addition, two research groups have mapped histone modifications (H3K4me1, H3K4me2, H3K4me3, H3K9Ac, H3K27Ac, and H3K27me3) in primary mouse B cells (50, 51). We tested whether any of these histone marks showed enrichment near the Ets1-binding peaks as compared to random chromatin. We found that peaks of H3K4me3 (associated with promoters) were strongly enriched on either side of the peak Ets1-binding locus (Figure 1E). Similarly, peaks of H3K4me2, H3K9Ac, and H3K27Ac (associated with both promoters and enhancers) were also strongly enriched flanking the Ets1-binding sites. Weaker enrichment of H3K4me1 (associated with enhancers) was also observed flanking Ets1-binding sites, while there was no enrichment of H3K27me3 (associated with repressed genes) near Ets1-binding peaks. None of the histone marks showed specific enrichment when random chromatin regions were used (not shown). These data indicate that binding of Ets1 is strongly associated with active promoters and enhancers of genes, but is associated weakly if at all with repressed sites.

**Figure 2:**
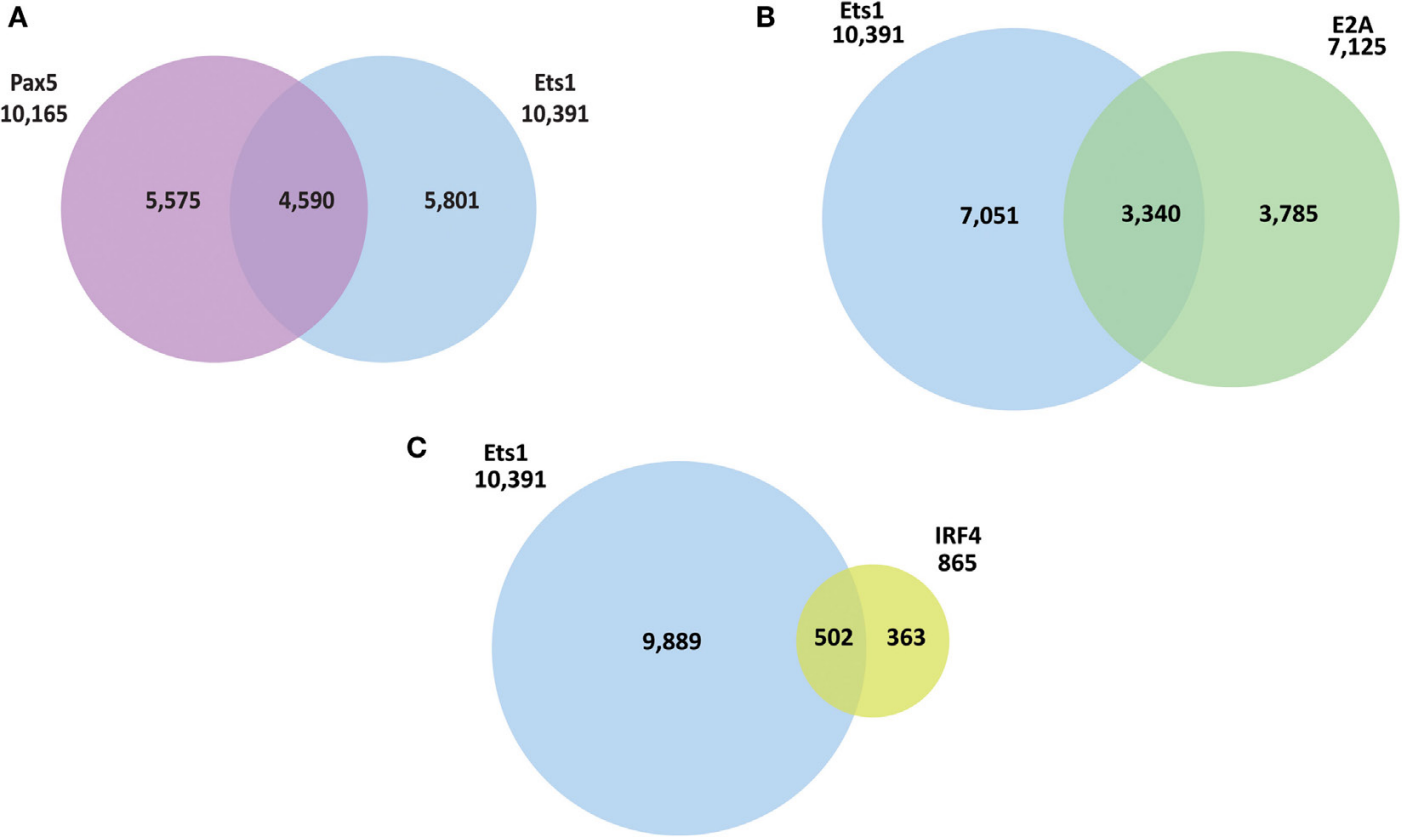
**Ets1-binding sites overlap with those of other B cell transcription factors**. Venn diagrams to show overlap between Ets1-bound regions in primary mouse B cells with **(A)** Pax5-bound regions, **(B)** E2A-bound regions, and **(C)** Irf4-bound regions. Pax5, E2A, and Irf4 ChIP-seq data come from studies described in Ref. (51, 53, 54).

ChIP-seq datasets are also available for Pax5 in mouse mature splenic B cells (51). We compared the binding sites of Pax5 with those bound by Ets1 to identify genes that might be co-regulated. There were 4,590 overlapping peaks common between Ets1 and Pax5 (Figure 2A), representing ~44% of all Ets1-bound regions and ~45% of all Pax5-bound regions. Among the genes having peaks for both Ets1 and Pax5 are some that encode genes that are crucial for B cell biology including CD79a, ICOS ligand, Foxp1, PLCγ2, and Vav1. Ontology enrichment analysis of the identified common targets of Ets1 and Pax5 indicate that they are associated with immune system activation and effector processes (not shown). This suggests that there is a strong overlap in function of Ets1 and Pax5 in B cells.

Binding sites for Tcf3 (E2A) (53) and Irf4 (54) have also been mapped in primary B cells. We found that ~47% of E2A-binding sites overlap with those of Ets1 (Figure 2B). These overlapping binding sites include genes such as *Cd19, Blnk*, and *Rasgrp1*. With Irf4, relatively few (865) binding sites were detected in primary naïve B cells (Figure 2C). However, of these 865 sites, ~58% overlap with Ets1-binding sites, including sites in important B cell genes such as *Cxcr4, Cxcr5, Lyn, Pax5, Ebf1*, and *Foxo1*. Overall, these studies show that Ets1 is targeted to promoters and/or enhancers of genes important for B cell activation and differentiation and can potentially co-regulate such genes along with other B cell transcription factors like Pax5, E2A, and Irf4.

### Identification of Changes in Gene Expression in the Absence of Ets1

**Figure 3:**
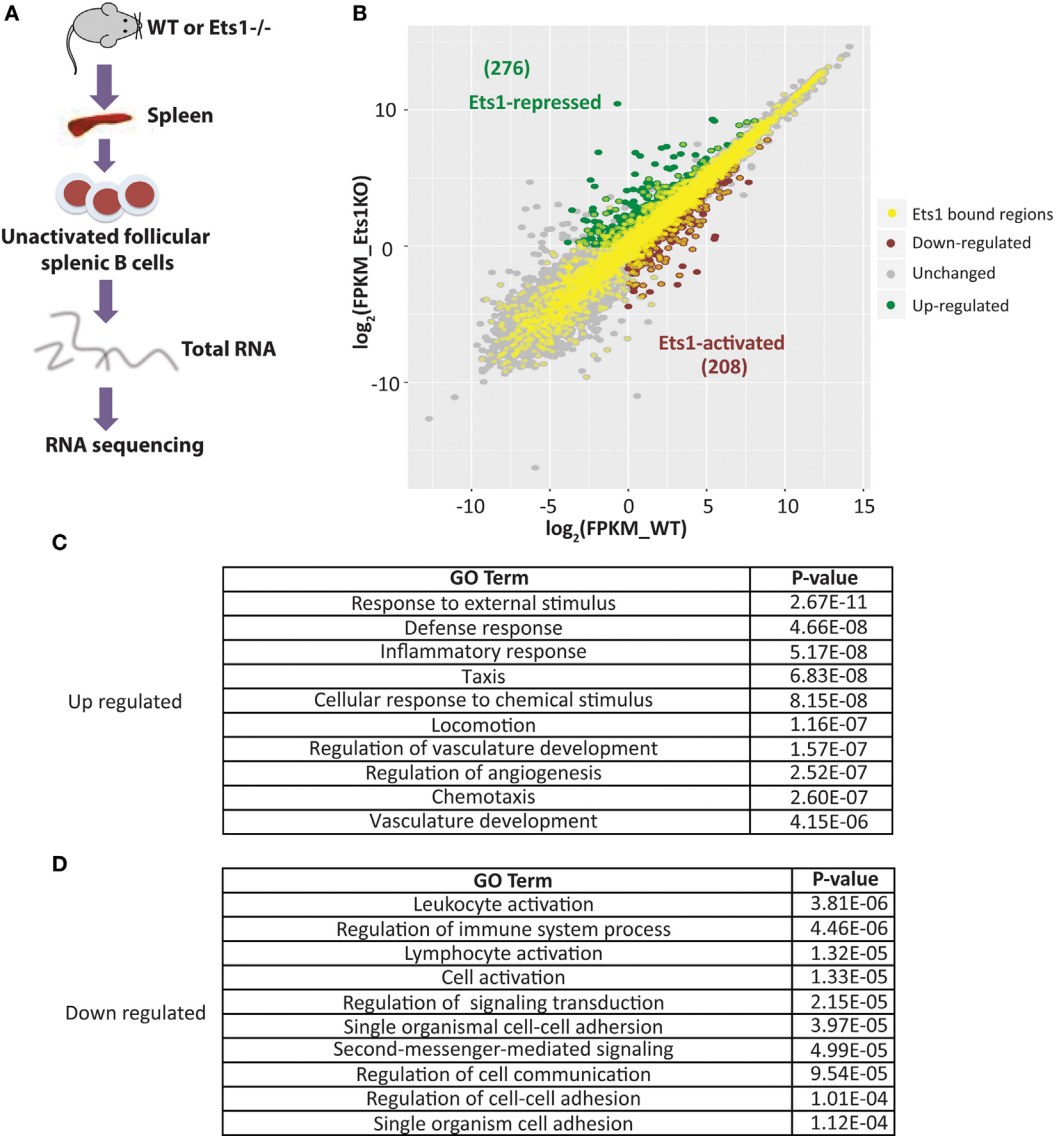
**Identification of genes whose expression in B cells requires Ets1**. **(A)** Scheme to isolate naive follicular B cells from the spleens of wild-type (WT) and *Ets1^−/−^* mice (*n* = 2 samples/genotype). **(B)** Scatterplot analysis of differential gene expression in WT and *Ets1^−/−^* B cells. Genes that are upregulated (Ets1-repressed) in *Ets1^−/−^* cells are shown as green dots, while genes that are downregulated (Ets1-activated) are shown as red dots. Gray dots are genes whose expression does not change and/or whose expression is less than 1.0 FKPM in both cell types. Dots with yellow centers are the genes that show an associated Ets1-binding site by ChIP-seq. Pathway analysis of genes repressed **(C)** and activated **(D)** by Ets1. The top 10 GO terms ranked according to *p*-value are shown for both Ets1-repressed and Ets1-activated genes.

The presence of a binding site for a transcription factor does not always correlate with its ability to activate or repress transcription from the gene. To better identify genes in B cells whose expression directly depends on Ets1, we performed RNA-sequencing using RNA isolated from WT and *Ets1^–/−^* splenic B cells (GEO Dataset: GSE83797). One potential complicating factor is that *Ets1^−/−^* mice have different B cell composition in the spleen, because they lack marginal zone B cells and their follicular B cells (1) have a more activated phenotype, (2) undergo altered isotype-switching, and (3) differentiate at higher rates to ASCs than their WT counterparts (23, 25, 26, 36, 67). Therefore, to compare similar populations of B cells from WT and *Ets1^−/−^* mice, we used flow cytometry to isolate follicular B cells (B220^+^ CD23^hi^ CD21^low^) that were not activated (CD80^neg^) and had not undergone class-switching (IgA^neg^ IgE^neg^ IgG1^neg^ IgG2a^neg^ IgG2b^neg^ IgG3^neg^) (Figure 3A). We avoided sorting B cells based on surface levels of IgM or IgD, because we did not want to activate the cells by cross-linking the antigen receptor. Total RNA was prepared from sorted B cell subsets from each genotype and subjected to deep sequencing.

We identified a list of genes that showed at least a twofold change in expression between WT and *Ets1^−/−^* B cells with a *q*-value of 0.05 or less and with a FPKM value of at least 1.0 in either genotype. Using these criteria, 484 genes showed altered expression in the absence of Ets1 (Figure 3B). Of these, the expression of 208 genes (43%) was downregulated in the absence of Ets1, suggesting that Ets1 activates the expression of those genes. The expression of 276 genes (57%) was upregulated in the absence of Ets1, suggesting that Ets1 might repress these genes (Figure 3B). Overall, the changes in expression of potential Ets1 target genes were mostly moderate (typically in the range of twofold to fourfold differences between WT and *Ets1^−/−^*). Unexpectedly, we found that the Pax5 gene was not one of those genes whose expression was altered in the absence of Ets1 (Figure S5 in Supplementary Material). Functional annotation of the molecular pathways that are overexpressed in *Ets1^−/−^* B cells (i.e., pathways repressed by Ets1) showed that they were predominantly associated with inflammatory responses and chemotaxis (Figure 3C; Figure S6A in Supplementary Material). In contrast, the molecular pathways that are under-expressed in *Ets1^−/−^* B cells (pathways activated by Ets1) were associated with immune cell signal transduction and activation (Figure 3D; Figure S6B in Supplementary Material). Thus, Ets1 is an important regulator of B cell functions by both positively and negatively regulating various aspects of the immune response.

### Genes Implicated in Autoimmunity Are among Those Controlled by Ets1

**Figure 4:**
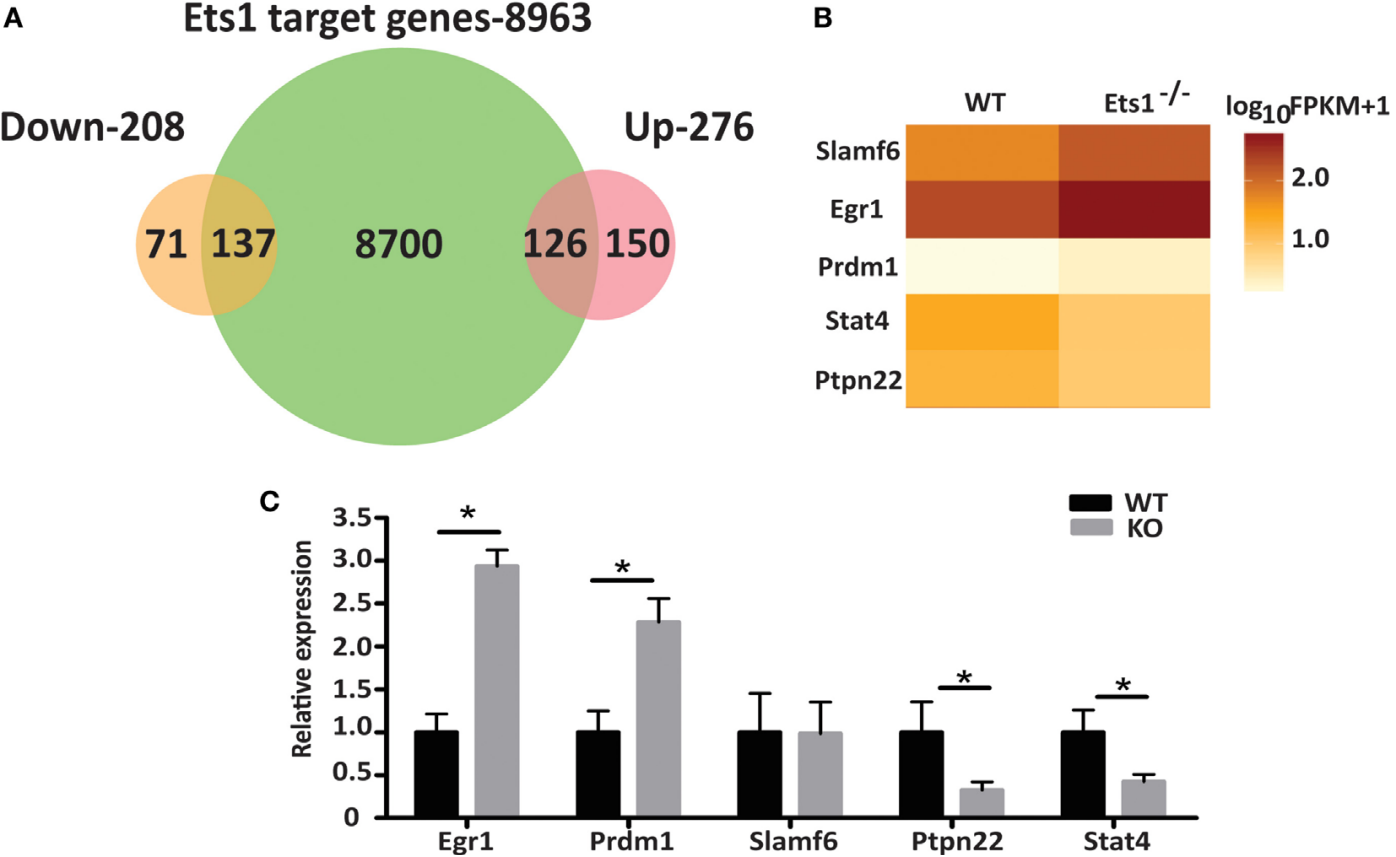
**Genes that are potential direct targets of Ets1**. **(A)** Venn diagram of overlap of ChIP-seq dataset with genes upregulated in the absence of Ets1 (276 genes) or downregulated in the absence of Ets1 (208 genes). **(B)** Heat map of selected Ets1 target genes associated with autoimmunity. **(C)** qPCR analysis of the expression of the target genes shown in part B (*n* = 4 for each genotype) using cDNA from sorted (B220^+^ CD23^hi^ CD21^low^ CD11b^neg^ CD80^neg^ IgA^neg^ IgE^neg^ IgG^neg^) follicular B cells. Shown is average ±SEM, *p < 0.05.

We compared the ChIP-seq dataset that contained binding sites for Ets1 to the RNA-seq dataset that showed which genes changed expression in *Ets1^−/−^* B cells to find genes that might be directly regulated by Ets1. There were 263 genes that contained a nearby Ets1-binding peak and showed an alteration in expression in the absence of Ets1 (Figure 4A). This represents ~3% of genes containing Ets1-binding peaks. Among these genes, 137 (~52%) are activated by Ets1 and 126 (~48%) are repressed by Ets1. Examining the list of 263 genes, we identified numerous putative Ets1 target genes where SNPs have been associated with autoimmune disease susceptibility (Table 1). Additional Ets1 target genes include ones important for immune function such as *Il5ra, Tlr1, Traf4*, and *Ltk*, but that not yet been implicated as susceptibility loci for autoimmune disease. In further studies, we focused on those genes listed in Table 1, because of their known associations with immune regulation and autoimmune disease susceptibility.

**Table 1:** **List of potential Ets1 target genes associated with autoimmune disease**.

Gene name	Change in *Ets1^−/−^* B cells	Autoimmune diseases linked to single-nucleotide polymorphisms in the gene (reference)	Function
Antxr2	Downregulated (4X)	Ankylosing spondylitis (68)	Membrane protein-binding extracellular matrix; unknown function in B cells
Ccdc88b	Downregulated (2X)	Sarcoidosis (69)	Coiled-coil domain protein of unknown function in B cells
Cr2	Downregulated (2.3X)	Systemic lupus erythematosus (70)	Receptor on B cells that binds complement fragment C3d
Gimap4	Downregulated (2.2X)	Behcet’s disease (71)	GTPase of unknown function in B cells
Ifi30	Upregulated (2.2X)	Multiple sclerosis (72)	Enzyme involved in antigen processing in B cells
Il6ra	Downregulated (2.3X)	Rheumatoid arthritis, type I diabetes (73)	Receptor for cytokine IL-6 involved in stimulating B cell proliferation and antibody-secreting cells (ASCs) formation
Itgam	Upregulated (3.3X)	Systemic lupus erythematosus (74); systemic sclerosis (75)	Integrin subunit involved in regulating BCR signaling
Nod2	Upregulated (5.2X)	Psoriasitic arthritis (76); Behcet’s disease (77)	Receptor for bacterial products like muramyl dipeptide; triggers NFκB activation in B cells
Pdcd1lg2	Downregulated (2.2X)	Ankylosing spondylitis (78)	Membrane-bound ligand for the PD-1 receptor that inhibits T cell activation
Pde2a	Downregulated (4X)	Rheumatoid arthritis (79)	Enzyme that degrades cAMP; unknown function in B cells
**Prdm1**	Upregulated (2.5X)	Systemic lupus erythematosus (80); rheumatoid arthritis (81)	Transcription factor driving ASC formation
**Ptpn22**	Downregulated (2.1X)	Type I diabetes (82); rheumatoid arthritis (83); systemic lupus erythematosus (84); antineutrophil cytoplasmic antibody-associated vasculitis (85); psoriatic arthritis (86); Myasthenia gravis (87); Graves’ disease (88)	A phosphatase that can regulate BCR signaling; variably reported to either enhance or suppress B cell responses
**Slamf6**	Upregulated (2.8X)	Graves’ disease (89)	Membrane receptor implicated in controlling B cell tolerance to self-antigens
**Stat4**	Downregulated (2.9X)	Behcet’s disease (90); rheumatoid arthritis (91); systemic lupus erythematosus (92); psoriasis (93); Sjogren’s syndrome (94); systemic sclerosis (95)	Transcription factor-mediating IL-12 signaling in B cells
Tlr1	Downregulated (4.6X)	Type I diabetes (96); Graves’ disease (97); alopecia areata (98)	Receptor with TLR2 to form a receptor for bacterial triacyl lipopeptide
Traf1	Downregulated (4.9X)	Rheumatoid arthritis (91); systemic lupus erythematosus (99); alopecia areata (100)	Signal transduction molecule that can associate with various TNF receptor family members including CD40, OX40, BCMA, and TACI
Zc3h12c	Upregulated (2.1X)	Psoriasis (101)	Transcription factor that inhibits inflammatory cytokine production
Zmiz1	Downregulated (2.7X)	Vitiligo (102); psoriasis (103)	PIAS-like co-regulator protein that is involved in regulating activity of transcription factors like p53 and Smads

*Genes noted in bold text are those which we follow-up on further in this report*.

We selected a subset of potential Ets1 target genes and designed primers to test their expression in qRT-PCR. The genes selected for further analysis were *Egr1, Stat4, Prdm1, Slamf6*, and *Ptpn22* as shown in Figure 4B and Figure S7 in Supplementary Material. qRT-PCR analysis showed that *Egr1, Stat4, Prdm1*, and *Ptpn22* all displayed a pattern of expression consistent with the RNA-seq results (Figure 4C). On the other hand, expression of *Slamf6* did not appear to change in the absence of Ets1 in this analysis.

Stat4 and Ptpn22 showed decreased expression in the absence of Ets1, while Egr1 and Prdm1 are elevated. Therefore, Ets1 may promote the expression of Stat4 or Ptpn22, while suppressing expression of Egr1 or Prdm1. Alternatively, these changes in Stat4, Ptpn22, Egr1, and Prdm1 may instead represent secondary changes in gene expression that reflect the fact that *Ets1^–/−^* B cells are primed to become activated and differentiate to ASCs, rather than being important drivers of the *Ets1^–/−^* B cell phenotype. If Ptpn22 or Stat4 were target genes that mediated the effects of Ets1 on B cell differentiation, then we would expect that expression of Stat4 and Ptpn22 in B cells might in part mimic the effects of expression of Ets1, such as its ability to suppress ASC generation. To test this, we cloned cDNAs encoding Stat4 and Ptpn22 into a retroviral vector so that we could restore expression of these genes to *Ets1^−/−^* B cells and overexpress them in WT B cells. WT or *Ets1^−/−^* splenic B cells were stimulated with LPS and infected with control virus (MIGR1), virus-expressing Ets1 (MIGR1-Ets1), or viruses-expressing Stat4 or Ptpn22 (MIGR1-Stat4 or MIGR1-Ptpn22). We monitored cellular differentiation to ASCs (B220^low^ CD138^+^) using flow cytometry in the GFP^+^ virally infected cells.

**Figure 5:**
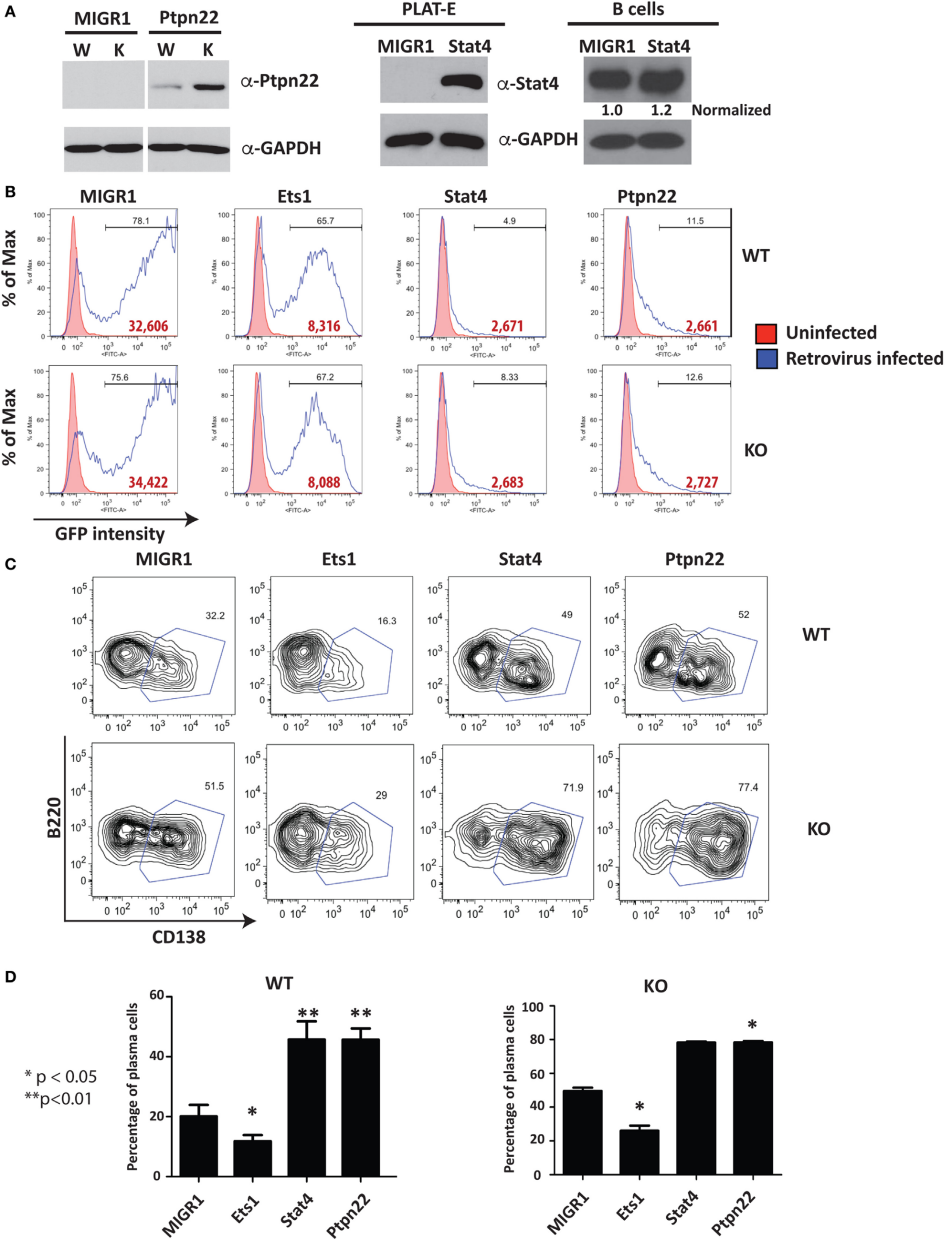
**Restoration of selected target genes in *Ets1^−/−^* B cells**. **(A)** Western blot analysis of Ptpn22 [with lysates from unsorted virally infected wild-type (W) and knockout (K) B cells] and Stat4 expression [with lysates from the packaging cell line (PLAT-E) or virally infected B cells (B cells); note that the small text under the Stat4 blot represents Stat4 expression levels normalized to GAPDH]. **(B)** Green fluorescent protein (GFP) profiles of virally infected cells. Red shaded curves are background staining of control non-infected cells, while open black curves are GFP staining in virally infected cells. Bold red type is the mean fluorescent intensity of GFP in the GFP^+^ gated population. **(C)** Analysis of GFP^+^ WT and *Ets1^−/−^* B cells to show differentiation to B220-low CD138^+^ plasmablasts (boxed). **(D)** Mean ± SEM of the percentage of plasmablasts within the GFP^+^ population in three independent viral infection experiments.

Western blot using extracts of virally infected B cells showed that B cells infected with Ptpn22 virus overexpress Ptpn22 (Figure 5A). Retrovirally expressed Stat4 was also easily detectable in the packaging cell line (Figure 5A), but its overexpression in B cells was less obvious due to the high levels of endogenous Stat4 in this cell type (Figure 5A). Viruses encoding Stat4 or Ptpn22 reproducibly resulted in low percentages of GFP^+^ virally infected B cells (5–10%) and low intensity of GFP staining, while empty virus or virus encoding Ets1 resulted in high percentages (40–80%) of GFP^+^ cells and high intensity of GFP staining (Figure 5B). Expression of Ets1 suppresses development of B220^lo^CD138^+^ plasmablasts compared to B cells infected with empty vector (MIGR1) (Figure 5C). On the other hand, we found that there was a dramatic increase in the B220^lo^CD138^+^ plasmablast cell numbers in B cells expressing Stat4 or Ptpn22 (Figure 5D), indicating that these genes stimulate the development of ASCs.

**Figure 6:**
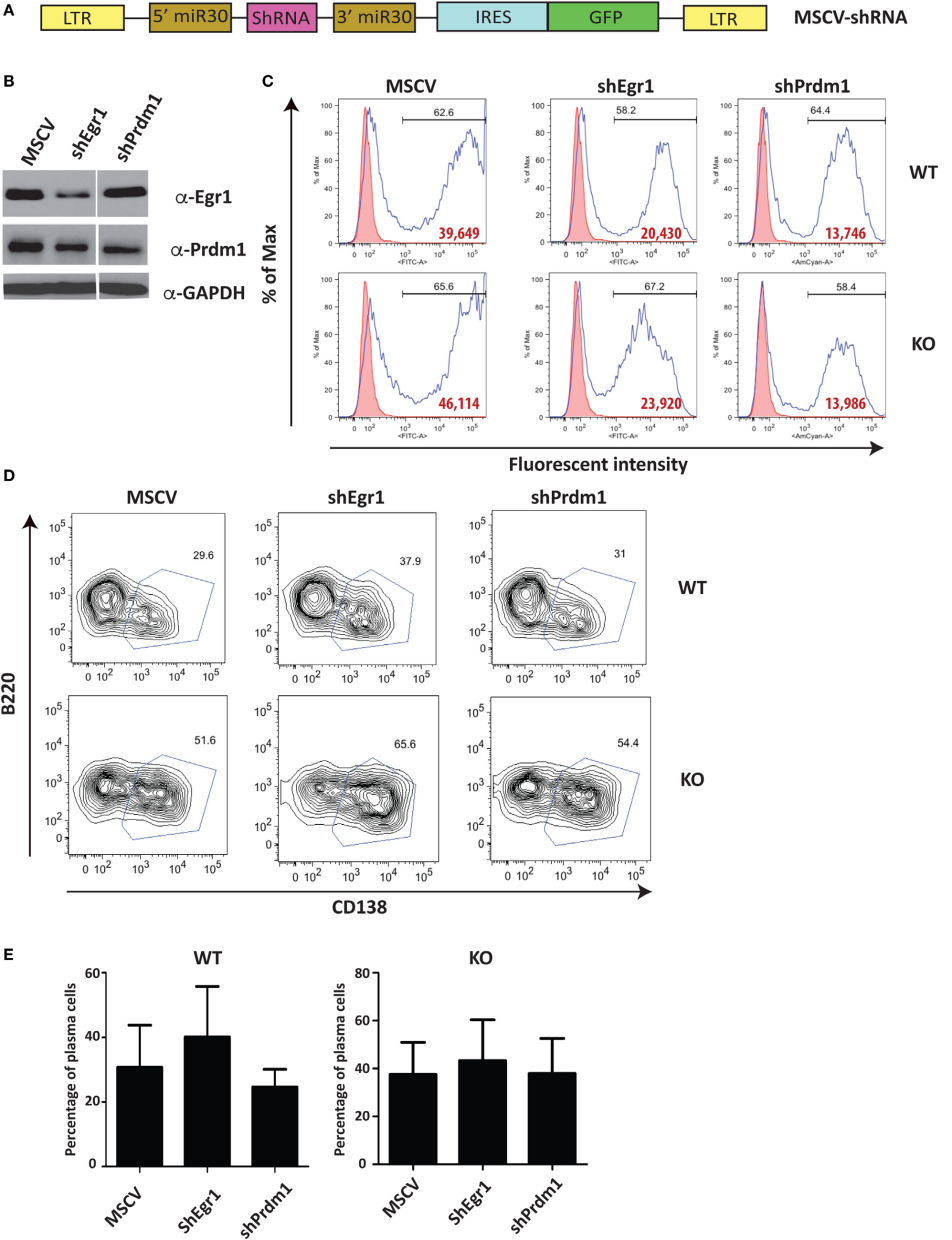
**Reduction of selected target genes in *Ets1^−/−^* B cells**. **(A)** Diagram of the retroviral construct encoding shRNAs used for knock down of gene expression in B cells. **(B)** Egr1 and Blimp1 expression analyzed by Western blot in lysates of B cells infected with shRNA viruses. **(C)** Green fluorescent protein (GFP) (MSCV and Egr1) and Ametrine (Prdm1) profiles of virally infected cells. Bold red type is the mean fluorescent intensity of GFP (or Ametrine) in the GFP^+^ (or Ametrine^+^) gated population. **(D)** B220 versus CD138 staining in the GFP^+^ (or Ametrine^+^) cells to show differentiation to B220-low CD138^+^ plasmablasts (boxed). **(E)** Mean ± SEM of the percentage of plasmablasts within the GFP^+^ (or Ametrine^+^) population in three independent viral infection experiments.

**Figure 7:**
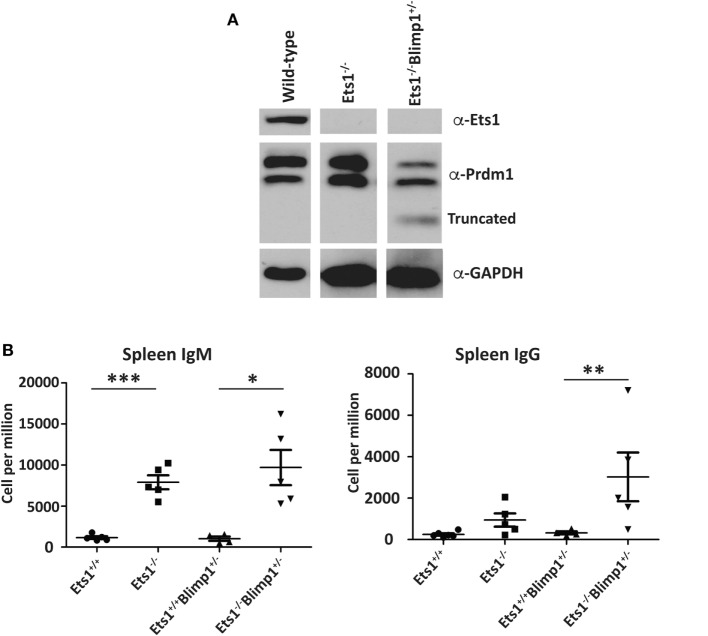
**Reduction of selected target genes in *Ets1^−/−^* B cells**. **(A)** Analysis of Blimp1 expression in lipopolysaccharide-stimulated *Ets1^+/+^, Ets1^−/−^*, and *Ets1^−/−^Prdm1^+/gfp^* B cells. The location of the full-length wild-type and the truncated proteins is indicated on the panel. **(B)** ELISPOT analysis of the numbers of IgM- and IgG-secreting cells in the spleens of unchallenged mice of the indicated genotypes (*n* = 4–5 for each genotype). *p < 0.05, **p < 0.01, and ***p < 0.001.

Unlike Stat4 and Ptpn22, Egr1 and Prdm1 were upregulated in *Ets1^−/−^* B cells by about twofold to threefold, suggesting that Ets1 represses expression of these genes. We used a retroviral vector encoding shRNAs to knockdown expression of these genes in B cells (Figure 6A) (59). Expression of shRNA against Egr1 was effective in reducing levels of Egr1 protein in stimulated B cells (Figure 6B), but did not impair formation of B220^low^CD138^+^ plasmablasts (Figures 6C–E). The Prdm1 shRNA was also able to knockdown expression, although it was less efficient in *Ets1^−/−^* B cells (Figure 6B and data not shown). The Prdm1 shRNA did not alter ASC differentiation in WT or *Ets1^−/−^* B cells (Figures 6C–E), despite the fact that Blimp1 is known to be essential for plasma cell generation. This is likely because sufficient Blimp1 is still expressed to allow ASC differentiation. To further assess the role of Prdm1 in the phenotype of *Ets1^−/−^* B cells, we crossed *Ets1^−/−^* mice to mice carrying a GFP knock in allele in the *Prdm1* locus that disrupts expression of the *Prdm1* gene and leads to reduced levels of full-length functional Blimp1 protein (104). Homozygous *Prdm1* knockout mice carrying this allele die embryonically due to a combination of developmental defects, but heterozygous mice are viable (104). We generated *Ets1^−/−^Prdm1^gfp/+^* mice that carry a single copy of *Prdm1* and express reduced levels of Blimp1 (Figure 7A). The numbers of ASCs in these mice and control mice was quantitated using ELISPOT, which showed that reduced levels of *Prdm1* was not sufficient by itself to restrain excess ASC formation in the absence of Ets1 (Figure 7B). In summary, the changes in expression of the genes we tested (*Stat4, Ptpn22, Egr1* and *Prdm1*) cannot by themselves explain the effects of Ets1 on ASC formation.

## Discussion

Over time, evidence has accumulated that Ets1 is a key regulator of B cell differentiation and that reduced levels of Ets1 are associated with the development of autoimmune diseases. We previously demonstrated that Ets1 controls B cell differentiation in part *via* the ability of Ets1 protein to directly bind to the Blimp1 protein and prevent Blimp1 interaction with DNA (22, 29). However, it remains unclear whether this protein–protein interaction is the main mechanism by which Ets1 inhibits B cell differentiation into ASCs or not. Because Ets1 is a transcription factor, it is important to understand what genes Ets1 regulates in B cells and how they may contribute to the control of B cell differentiation and secretion of autoantibodies. In this study, we show that Ets1 binds to and is required for normal expression of ~260 genes in mature B cells, including a subset of genes highly linked to immune responses and autoimmune disease susceptibility.

A large percentage (~50%) of the Ets1-binding sites, we identified in primary B cells, are localized in proximal promoters of genes in regions enriched for histone marks associated with active promoters (H3K4me3, H3K4me2, H3K9Ac, and H3K27Ac). Such a high percentage of binding sites in the proximal promoter is not always the case in ChIP-seq analyses of transcription factors. For instance, a ChIP-seq study of PU.1 and SpiB binding in the WEHI-279 B cell line found only ~15% of binding sites localized in the proximal promoter (105). Similarly, analysis of Runx1 binding in pro-B cells found that only ~10% of sites were in the proximal promoter of target gene (106). On the other hand, ChIP-seq analysis of Ets1 binding in human hematopoietic and embryonic stem cell lines showed that Ets1 binding was enriched in the promoter regions of genes in these cell types (107), although in the same study Ets1 binding was fairly equally distributed between promoters and enhancers in mouse cell lines. In the human Jurkat T cell line, Ets1 was shown to bind redundantly to proximal promoters of housekeeping genes along with other members of the Ets gene family, but to bind specifically to distal T cell enhancers (108). We found a similar pattern in primary mouse B cells, where Ets1 tends to bind only to the promoter of genes that have housekeeping functions (not shown). However, when Ets1 binds to distal regulatory elements (e.g., enhancer sequences) or to both promoters and distal regulatory sequences, those genes tend to be involved in specific B cell immune response pathways.

We previously showed that Ets1 can bind to the Pax5 gene, transactivate the Pax5 promoter and sustain Pax5 expression in differentiating B cells (22, 29). Here we confirm using ChIP-seq that Ets1 binds to both the Pax5 promoter and to the intron 5 B cell-specific enhancer of Pax5. However, somewhat surprisingly the levels of Pax5 mRNA are not significantly changed in *Ets1^−/−^* B cells as compared to WT B cells, as detected by RNA-sequencing. Several explanations could account for this. First, other Ets family members expressed in B cells (such as Fli1, Spi1, Spib, Elk4, Elf1, Elf2, Gabpa, and/or Etv3) could bind to these sites in the absence of Ets1 and compensate to maintain Pax5 expression. Supporting a potentially redundant role for Ets family transcription factors in regulating Pax5 gene expression, the Ets protein PU.1 can also bind to Pax5 enhancer sequences (64, 109). Alternatively, Ets1, specifically, may be required to maintain Pax5 expression, but may only regulate the gene at certain stages of development or in response to certain stimuli. Finally, it is also possible that the Ets-binding sites at these regions might represent fortuitous binding without functional significance. Similar arguments may be made for the many other genes in which we detected Ets1 binding, but whose expression does not change in B cells lacking Ets1. For instance, we found that Ets1-binding sites were detected in many genes involved in the BCR signaling pathway. Yet, the expression of most of these genes was unchanged in the absence of Ets1. Indeed, only ~3% of genes with a nearby Ets1-binding site showed altered expression in mature Ets1 knockout B cells.

Interestingly, among the genes identified that had at least one Ets1-binding site nearby and whose expression changed in *Ets1^−/−^* B cells, we found many that have previously been implicated in immune responses and autoimmune disease susceptibility based on genome-wide SNP assays. We chose two genes whose expression is reduced in *Ets1^−/−^* B cells (Stat4 and Ptpn22) and restored their expression using retroviral vectors. An unexpected result of our study is that expression of either Stat4 or Ptpn22 has an opposite effect to the expression of Ets1 (i.e., Ets1 expression suppresses LPS-induced plasmablast formation, while Stat4 and Ptpn22 both promote this process). This observation is interesting in light of the fact that Stat4 and Ptpn22 are both strongly associated with susceptibility to multiple different autoimmune diseases (110, 111).

Stat4 is best known as a transcription factor activated downstream of IL-12 signaling where it plays an important role in the production of IFN-γ (112). In B cells, Stat4 and IL-12 signaling have been most closely linked to differentiation of B cells to B effector type 1 (Be1) cells that secrete IFN-γ (113). However, our results suggest that Stat4 has additional B cell-intrinsic effects that promote the formation of ASCs, since retrovirally induced production of Stat4 potentiated LPS-induced formation of B220^lo^CD138^+^ plasmablasts. This was true even though Stat4 was expressed at low levels in B cells using our retroviral construct. This effect of Stat4 may be independent of its roles in promoting IFN-γ production, although further studies are needed to confirm this. Recent data show that a lupus-associated SNP in the *STAT4* gene leads to increased Stat4 expression (114), supporting the idea that higher Stat4 expression may promote autoimmunity. Interestingly, knockout of the Stat4 gene reduces autoantibody production in lupus-prone B6.TC mice (115). These results are consistent with the data in this manuscript that show Stat4 expression is increased in B cells from autoimmune-prone *Ets1^−/−^* mice and that Stat4 expression in B cells potentiates development of ASCs.

We found that retroviral expression of Ptpn22 also potentiates formation of plasmablasts in response to LPS. In myeloid cells, Ptpn22 promotes TLR signaling by stimulating Traf3 autoubiquitination (116). It may play a similar role in B cells, resulting in increased LPS-derived signals that promote ASC generation. Altogether, our data indicate that Stat4 and Ptpn22 have opposite roles compared to Ets1 in regulating formation of ASCs in response to LPS stimulation. The low levels of Stat4 and Ptpn22 transcripts in *Ets1^−/−^* B cells may, therefore, be due to secondary changes in the cells in an attempt to compensate for excessive B cell activation and differentiation in the absence of Ets1.

We also investigated roles for two genes whose expression is upregulated in *Ets1^−/−^* B cells, *Egr1*, and *Prdm1*. Recently, B cells lacking Egr1 were shown to undergo reduced differentiation to CD138^+^ plasmablasts when cultured with LPS (117). Furthermore, Egr1 knockout mice secrete less antigen-specific antibody in response to immunization with a T-dependent antigen (117). Since Egr1 is overexpressed in *Ets1^−/−^* B cells, reducing the levels of this gene might be expected to reverse the phenotype of *Ets1^−/−^* B cells and reduce ASC numbers. We used shRNA to knockdown Egr1 in B cells undergoing differentiation and found that this did not change ASC generation in either WT or *Ets1^−/−^* B cells. Thus, Egr1 appears not to be an essential Ets1 target gene regulating B cell differentiation, but rather is likely upregulated as a secondary consequence of B cell activation.

Ets1-binding sites are found in the *Prdm1* locus and Ets1 has previously been suggested to directly repress transcription of the *Prdm1* gene in Th1-skewed T cells (118). Since Blimp1, the protein encoded by the *Prdm1* gene, is a key transcription factor driving ASC formation, it is reasonable to assume that the approximately twofold upregulation of Prdm1 in *Ets1^−/−^* B cells might explain their propensity to differentiate to ASCs. To test this, we used both knockdown of *Prdm1* in cultured B cells as well as gene-targeted mice. Knockdown of Prdm1 by shRNA did not impair ASC generation, potentially, because the knockdown did not fully eliminate Blimp1 expression. As an alternative approach to test whether reducing Blimp1 levels could restore normal ASC differentiation to mice lacking Ets1, we crossed *Ets1^−/−^* mice with mice carrying a heterozygous GFP knock in mutation in the Blimp1 locus that reduces Blimp1 expression by 50%. In the resulting *Ets1^−/−^Prdm1^gfp/+^* mice, Blimp1 levels in *Ets1^−/−^* B cells are normalized. Yet, these mice still show an excess of splenic ASCs. Therefore, Ets1-dependent repression of Blimp1 expression does not seem to be an essential mechanism for preventing ASC generation.

## Conclusion

Our study has provided a genome-wide list of target genes for the key B cell transcription factor Ets1. Given that normal expression of Ets1 is required to prevent autoimmune disease, it is interesting that a number of these Ets1 target genes are genes identified as susceptibility alleles of autoimmune diseases. We expect that our results will be helpful in gaining insight into the molecular mechanisms that contribute to autoimmune disease and the roles of B cells in this process.

## Availability of Data and Material

The datasets generated during the current study (RNA-seq and ChIP-Seq of Ets1 in B cells) are available in the GeoDatasets repository accession number GSE83797, https://www.ncbi.nlm.nih.gov/geo/query/acc.cgi?acc=GSE83797.

## Ethics Statement

Animal experiments were performed under the approval and guidance of the Institutional Animal Care and Use Committee (IACUC) of Roswell Park Cancer Institute protocol #UB1104M, “Ets Transcription Factors in Hematopoiesis.”

## Author Contributions

PS performed most experiments with help from AK. PS, SN, and LG-S wrote the manuscript. All authors read and approved the final version.

## Conflict of Interest Statement

The authors declare that the research was conducted in the absence of any commercial or financial relationships that could be construed as a potential conflict of interest.

## Supplementary Material

Figure S1 **Ets1-binding sites in the *Pax5* gene**. The genomic locus of the mouse *Pax5* gene (bottom) with Ets1-binding sites indicated as black bars along the top of the figure and by vertical columns of yellow shading. Note that Ets1 is strongly enriched at the promoter and in Intron 5 where a known B cell-specific enhancer is localized. Also shown are peaks of H3K27 acetylation and H3K4 monomethylation derived from the mouse ENCODE datasets, which mark active enhancers and promoters.

Figure S2 **Ets1-binding sites in the *Prdm1* gene**. The genomic locus of the mouse *Prdm1* gene (bottom) with Ets1-binding sites indicated as black bars along the top of the figure and yellow shaded vertical columns. Note that Ets1 is enriched in Intron 3 as well as sequences localized 50 kb downstream of the gene and sequences localized ~180 kb upstream of Prdm1.

Figure S3 **Ets1 binding sites in previously described target genes**. The genomic loci encoding *Nfkb1* (p50), *Cd79a* (Igα), and *H-2Aa* and *H-2Eb1* (MHC II isoforms), genes that have previously been described as Ets1 targets based on transient transfection assays. Each gene is shown in a format similar to that described for Figures S1 and S2 in Supplementary Material. Note that each gene contains Ets1-binding sites in the promoter and other nearby regions based on ChIP-seq, supporting the idea that these are true functional target genes of Ets1.

Figure S4 **Ets1-binding sites are found in a large percentage of genes involved in BCR signaling**. BCR signaling cascade analyzed by IPA software. Genes encoding proteins outlined in purple contain one or more Ets1-binding sites nearby.

Figure S5 **Expression of *Pax5* is not altered in Ets1-deficient B cells**. RNA-seq profiles for the *Ets1* gene, the *Pax5* gene, and a constitutively expressed housekeeping gene *Ppia* (peptidylprolyl isomerase A). Neither *Pax5* nor *Ppia* shows any change in transcription in *Ets1^−/−^* B cells. Overall, Ets1 transcription is also not reduced in *Ets1^−/−^* B cells, but the exons that are targeted in the knockout (part of the second exon and the third exon) show reduced/absent transcription.

Figure S6 **Heat maps to show expression changes of genes in certain selected pathways identified by gene ontology analysis**. Heat maps of the genes identified in the **(A)** defense response and **(B)** protein phosphorylation pathways showing clustering of wild-type and knockout B cells. Genes associated with autoimmune disease susceptibility that are further studied in this manuscript are highlighted by red type.

Figure S7 **Ets1-binding sites in the *Egr1, Stat4, Slamf6*, and *Ptpn22* genes**. The genomic loci of the mouse *Egr1, Stat4, Slamf6*, and *Ptpn22* genes with Ets1-binding regions shown along the top as black bars and as yellow shaded vertical columns. Also shown are peaks of H3K27 acetylation and H3K4 monomethylation derived from the mouse ENCODE datasets, which mark active enhancers and promoters.

## Abbreviations

ASCs, antibody-secreting cells; BCR, B cell receptor; ChIP-seq, chromatin immunoprecipitation-sequencing; ENCODE, encyclopedia of DNA elements; FPKM, fragments per kilobase of transcript per million mapped reads; GO, gene ontology; GWAS, genome-wide association study; IRES, internal ribosomal entry site; LPS, lipopolysaccharide; SNPs, single-nucleotide polymorphisms; ShRNA, short hairpin RNA.
