# Supplementary material for: Genome-Wide Identification of Target Genes for the Key B Cell Transcription Factor Ets1
Source: Front Immunol. 2017 Apr 7;8:383. doi: 10.3389/fimmu.2017.00383 (PMC5383717; doi:10.3389/fimmu.2017.00383)

Supplemental Figure 1

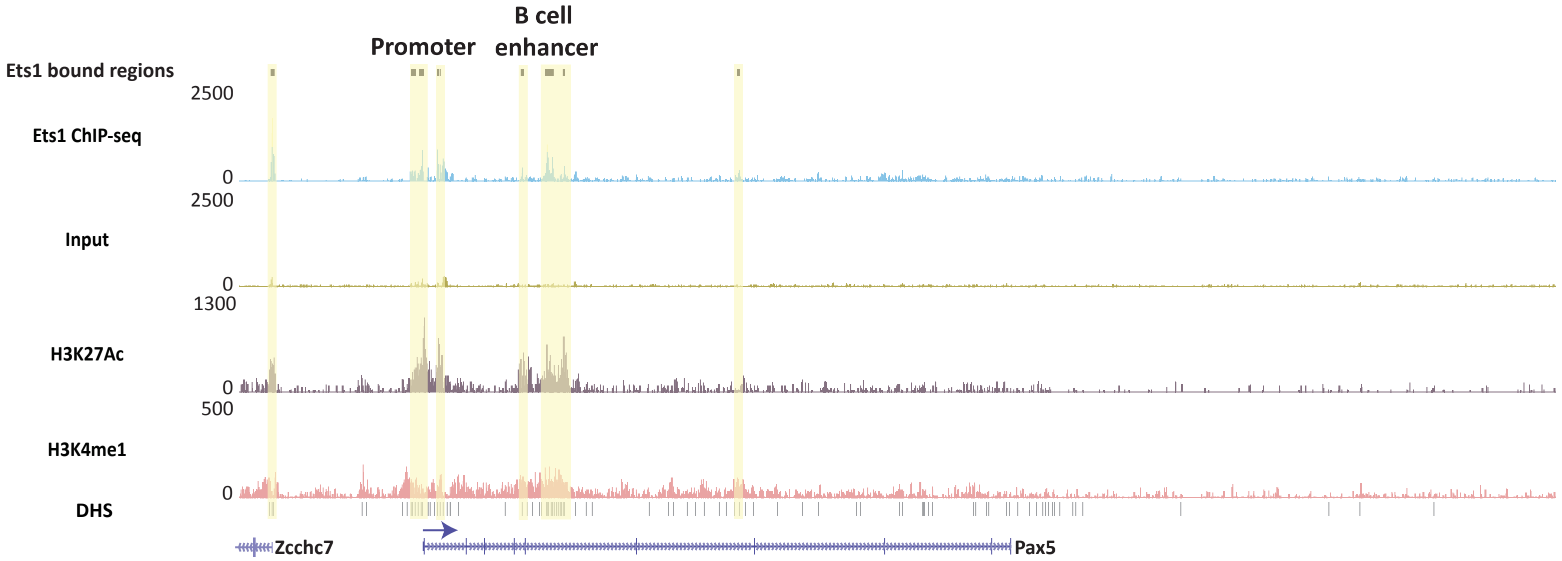

Supplemental Figure 2

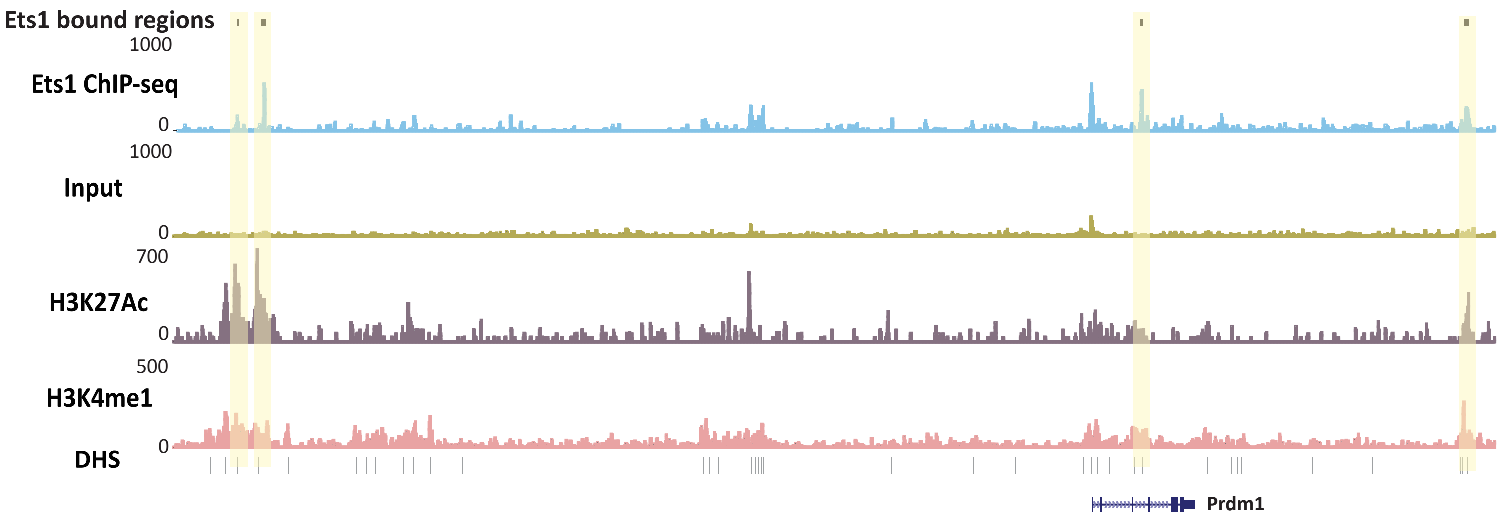

Supplemental Figure 3

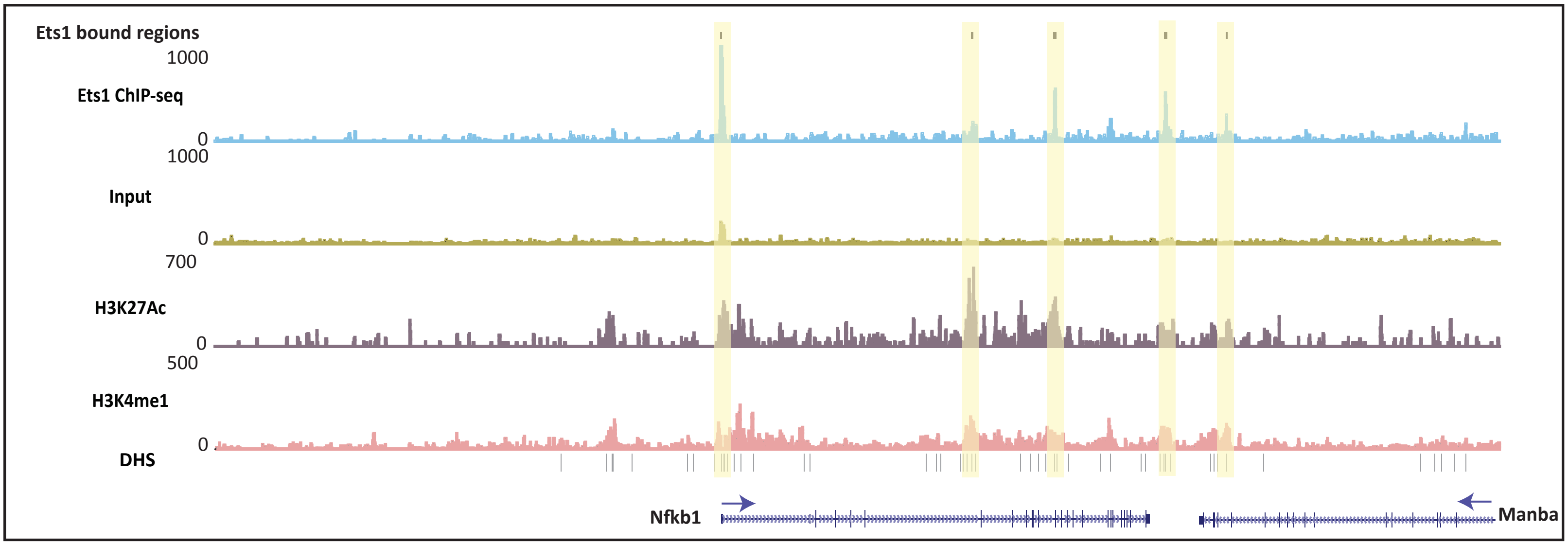

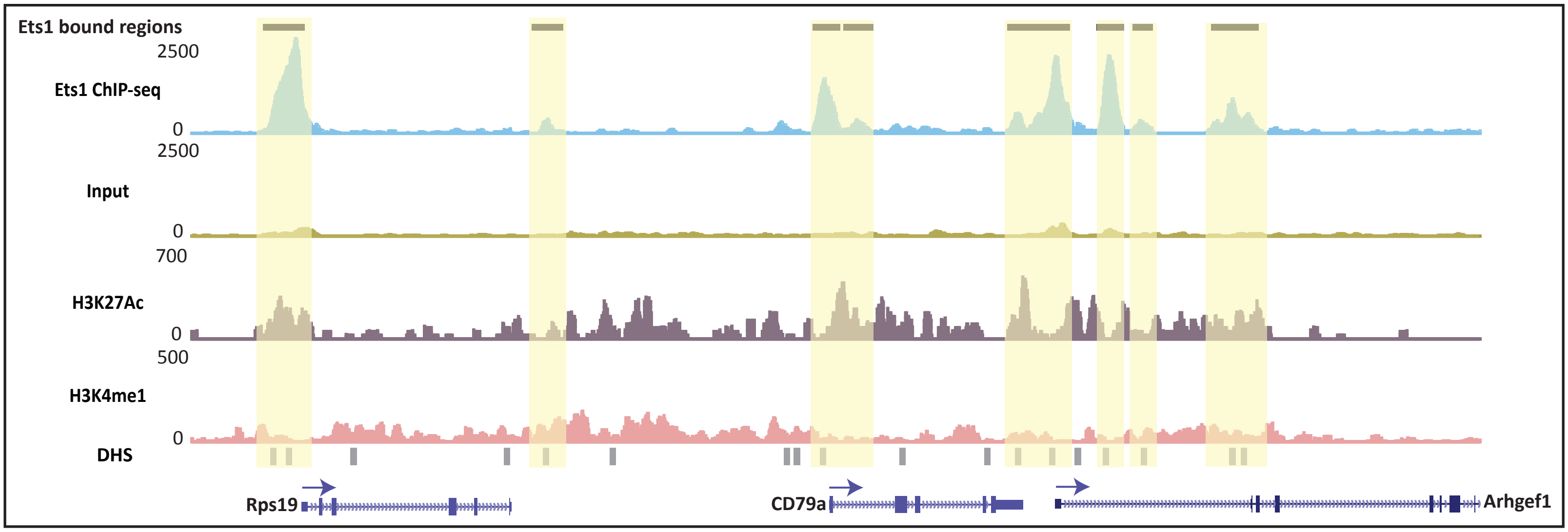

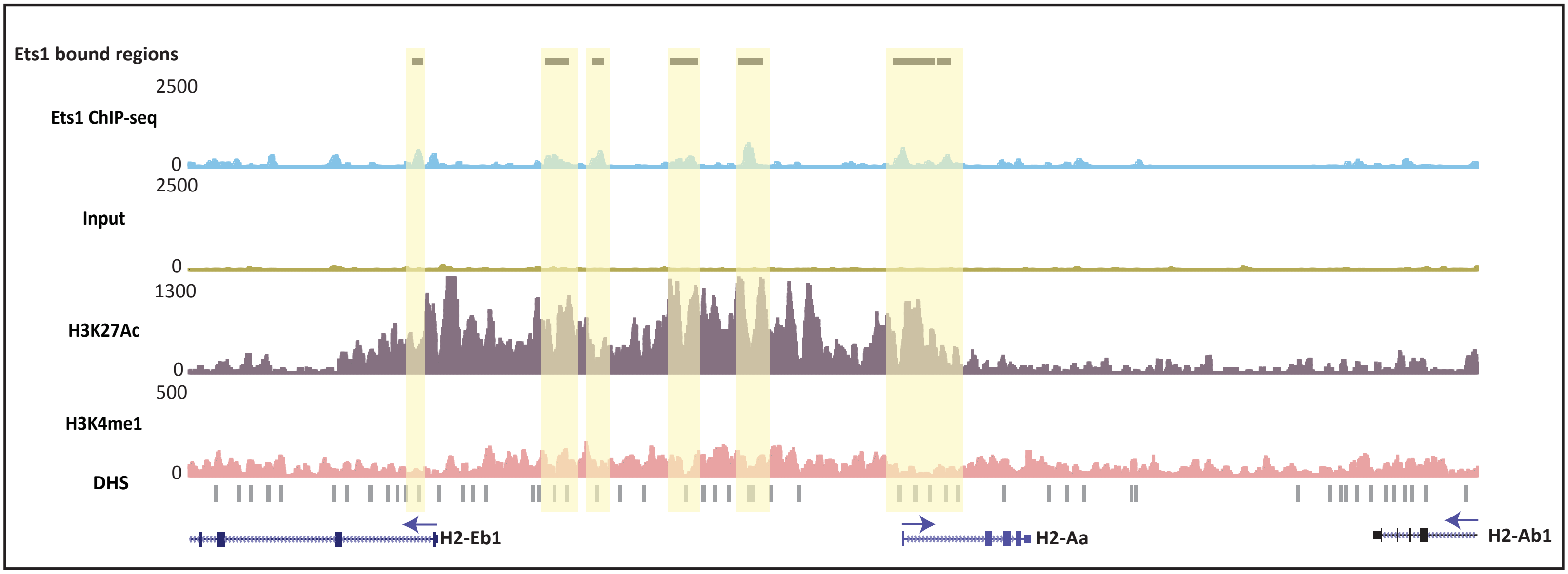

Supplemental Figure 4

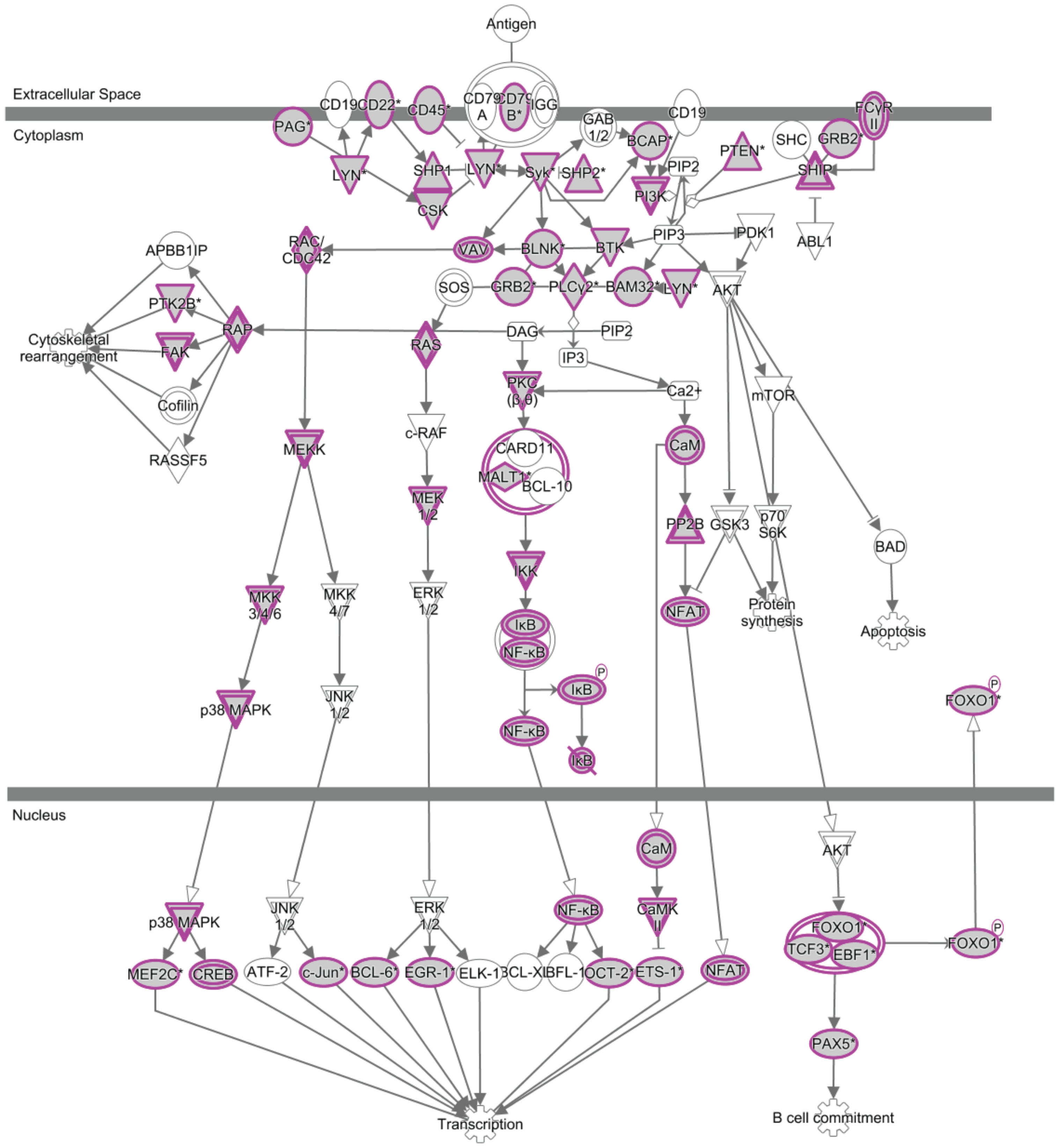

Supplemental Figure 5

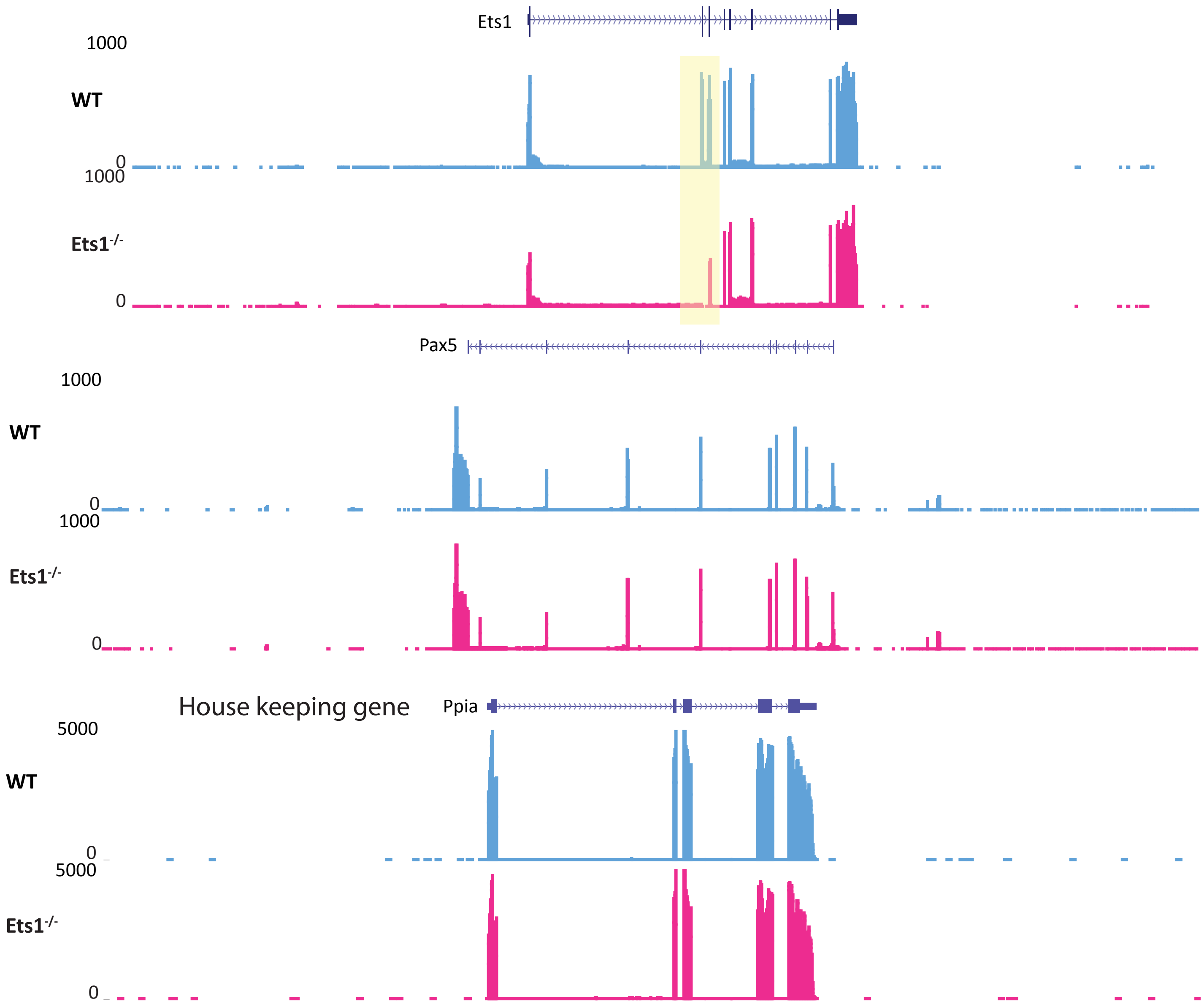

Supplemental Figure 6

A

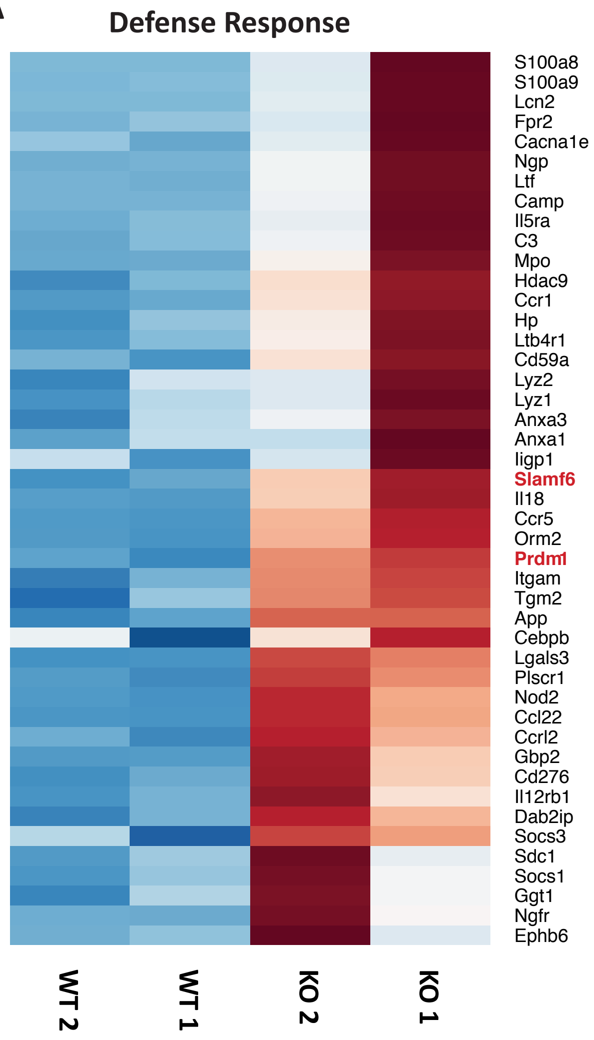

B

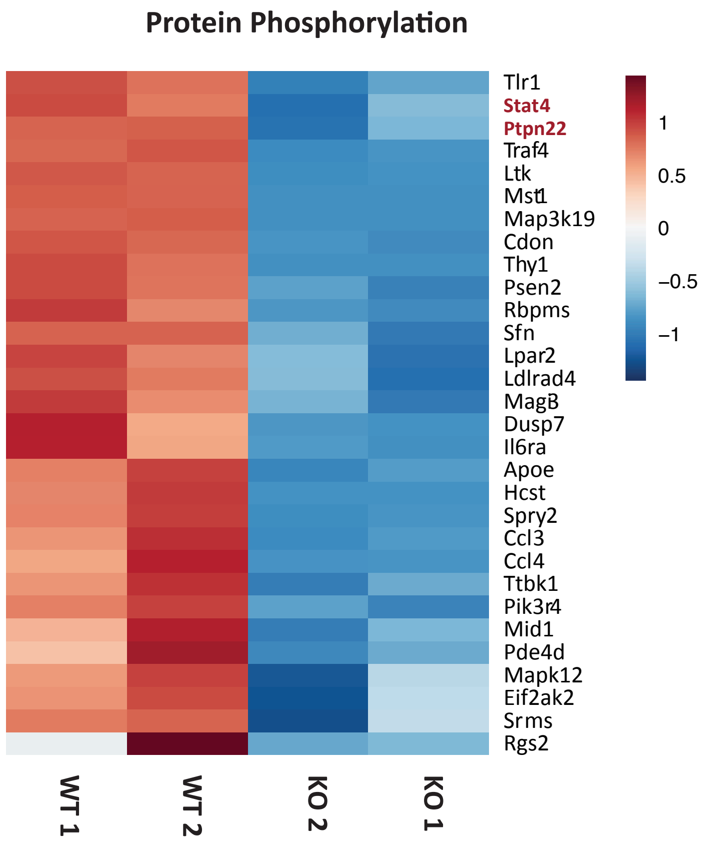

Supplemental Figure 7

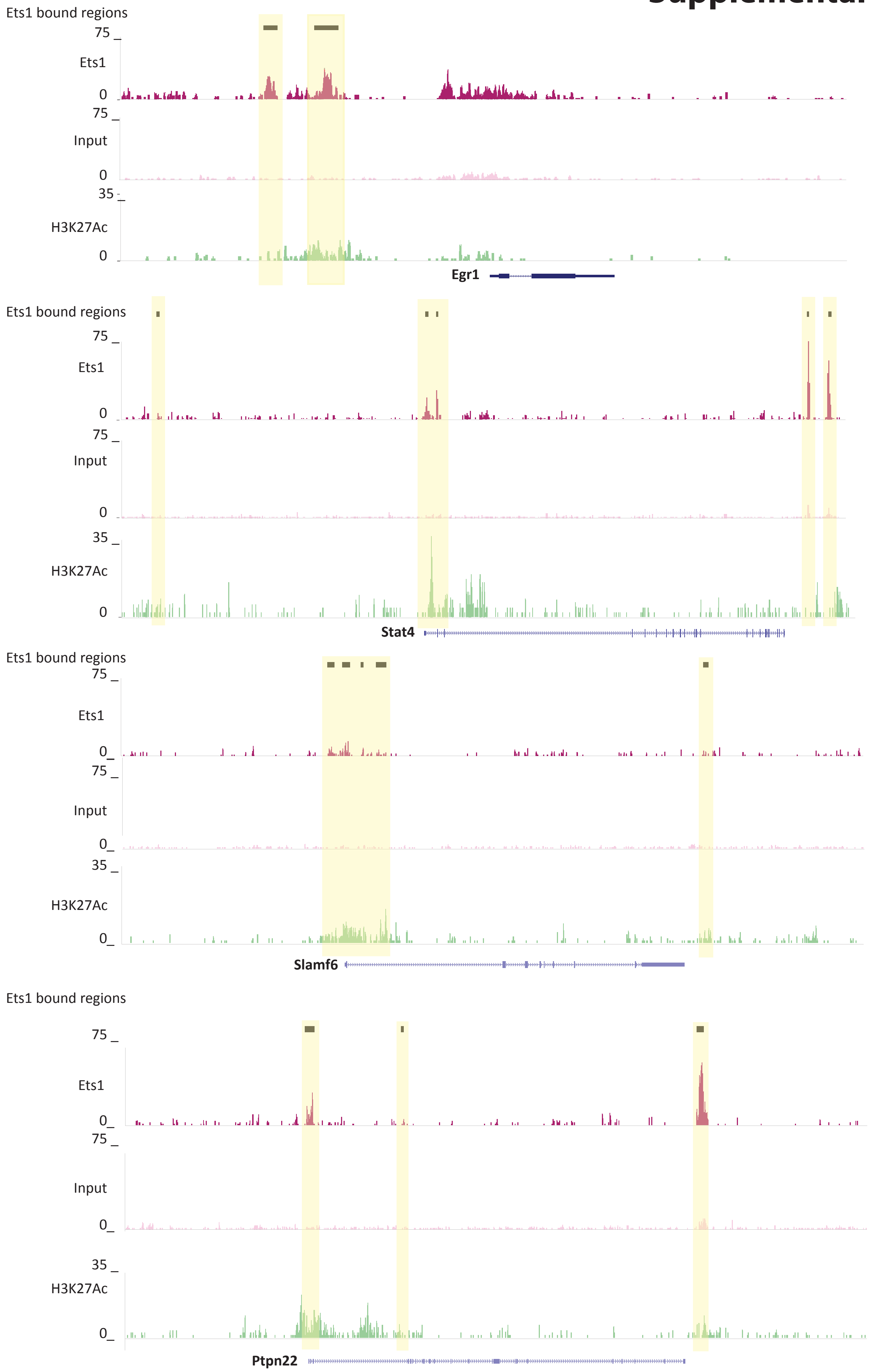

Supplement: Figure S1 — Ets1-binding sites in the Pax5 gene. The genomic locus of the mouse Pax5 gene (bottom) with Ets1-binding sites indicated as black bars along the top of the figure and by vertical columns of yellow shading. Note that Ets1 is strongly enriched at the promoter and in Intron 5 where a known B cell-specific enhancer is localized. Also shown are peaks of H3K27 acetylation and H3K4 monomethylation derived from the mouse ENCODE datasets, which mark active enhancers and promoters. [file Presentation_1.PDF]
